# Immunoprotective potential of BamA, the outer membrane protein assembly factor, against MDR *Acinetobacter baumannii*

**DOI:** 10.1038/s41598-017-12789-3

**Published:** 2017-09-29

**Authors:** Ravinder Singh, Neena Capalash, Prince Sharma

**Affiliations:** 1 Department of Microbiology, Panjab University, Chandigarh, India; 2 Department of Biotechnology, Panjab University, Chandigarh, India

## Abstract

*Acinetobacter baumannii* infections are responsible for major health problems in immunocompromised patients particularly in intensive care units. Due to rapid acquisition of and also inherent drug resistance, a vaccine is an effective treatment option against this pathogen. BamA, an outer membrane β-barrel assembly protein, was identified in *A. baumannii* as potential vaccine candidate by *in silico* analysis. The immunoprotective efficacy of this highly conserved protein was investigated against a virulent multidrug resistant clinical isolate using murine pneumonia model. Recombinant BamA elicited a high IgG antibody titer (160000) in mice. Opsonophagocytic killing assay showed non-neutrilizing, opsonizing antibodies with combinatorial bactericidal activity of antibodies and complement components. Active and passive immunization protected 80 and 60% mice respectively against intranasal challenge with lethal dose (10^9^ CFU) of virulent *A. baumannii* along with efficient clearance of bacteria in mice lungs and reduction in levels of pro-inflammatory cytokines viz. TNF-α, IL-6 and IL-1β in sera and lung tissue homogenate. Increase in levels of IL-10, an anti-inflammatory cytokine and reduction of neutrophils in lungs facilitated the control of infection. This study demonstrates the potential of BamA as effective vaccine candidate and a promising target for antibody-based therapy to protect against MDR *A. baumannii* infections.

## Introduction

*A. baumannii* is an opportunistic nosocomial pathogen that causes pneumonia, sepsis and soft tissue infections. Due to its ability to survive on dry surfaces and to form biofilms on biotic/abiotic surfaces, it has emerged as a critical pathogen. Currently, antibiotics are the only treatment option against these infections but the frequent use of antibiotics has led to emergence of multidrug resistant strains particularly against cephalosporins, fluoroquinolones, aminoglycosides and even carbapenems that has made treatment of *A. baumannii* infections complicated^1–5^. *bla*-NDM harboring strains are resistant to all broad spectrum β-lactams due to enzymatic degradation by β-lactamases whereas resistance to broad spectrum cephalosporins usually results from over-expression of the AmpC-type cephalosporinase gene and from acquisition of extended-spectrum β-lactamases (ESBLs)^2,6,7^. Due to failure of these antibiotics, use of colistin against *A. baumannii* has been increased that has unfortunately led to the emergence of colistin-resistant strains^4,8^. Recently, WHO has released a list of antibiotic resistant priority pathogens and mentioned *A. baumannii* as the critically dangerous pathogen^9^. Emergence of multi-, extensive- and pan-drug resistant strains worldwide is a serious concern and novel approaches are direly needed to prevent *A. baumannii* infections in which effective vaccination could become efficient and economical method to prevent the bacterial infections.

Numerous vaccine candidates have been tested against this ubiquitous opportunistic pathogen. Immunization with whole cell organism^10^, outer membrane vesicles^11^, outer membrane complex^12^, capsule components^13^ or poly-N-acetyl-β-(1–6)-glucosamine^14^ has been suggested as efficient vaccination approach due to abundance of immunogenic components in them. Current immunization strategies target a single or multiple outer membrane proteins and such antigens are easy to prepare and are safe as there is no risk of pathogen reverting back to its virulent form and there are few adverse effects as compared to live attenuated or killed whole cell vaccination. Outer membrane proteins Ata^15^, OmpA^16^, OmpK, Ompp1 and FKIB^17^ provided partial to complete mice protection against bacterial challenge in laboratory trials but still, a marketable vaccine against virulent, MDR *A. baumannii* is so far not available. In our previous work, immunization with a putative pilus assembly protein, FilF^18^ and outer membrane nuclease^19^ were found conferring partial protection against *A. baumannii* ATCC 19606 and controlled the infection by reducing bacterial load in organs and pro-inflammatory cytokine levels. Outer membrane β-barrel assembly protein, BamA was predicted as a potential vaccine candidate *in silico*. Although BamA is not characterized in *A. baumannii* but it is highly conserved among the Gram negative bacteria due to its crucial role in outer membrane protein assembly. BamA is the surface exposed protein from *bam* operon and its structure is well described in *E. coli*.^20^.

Outer membrane proteins act as effective protective antigens because they are capable of inducing host immune system and providing protection against bacterial challenge^16–19,21,22^. In this work, we showed that BamA is highly conserved in *A. baumannii* and has the ability to evoke immune response in mice leading to efficient immunoprotection.

## Results

### *In silico* prediction of BamA as vaccine candidate

Vaxign predicted BamA a potential vaccine candidate as it is an outer membrane protein as predicted by PSORTb (*p* > 0.9) with high adhesion probability, no trans-membrane helices and complete dissimilarity with human and mouse proteomes (Suppl. Table 1). BamA consists of bacterial surface antigen domains and outer membrane protein assembly domain. N-terminal of BamA contains POTRA domains which are responsible for interaction of BamA with other proteins in BAM complex (Suppl. Figure 1).

**Figure 1:**
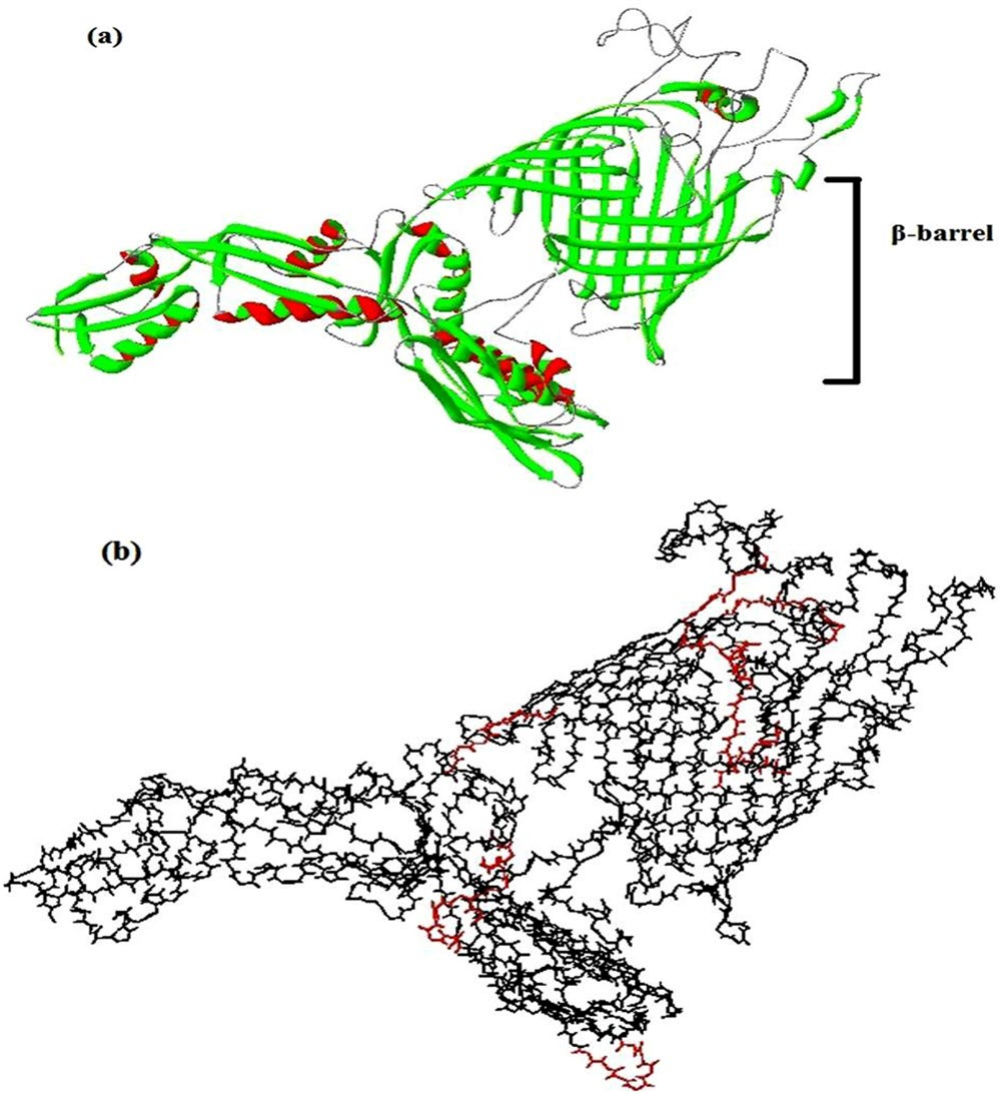
(**a**) Three dimensional structure of BamA modelled using automated homology prediction with SWISS-MODEL server based on the template FhaC (a member of the Omp85 transporter superfamily) (PDB ID: 2QDZ) which is a β-barrel protein in *Bordetella pertussis*. Overall quality factor of modelled structure was 74.161 with g-factor and Z-score of 0.43 and -6.9 respectively. Ramachandran plot showed 771 residues in favoured, 32 in allowed and 8 in outlier region that demonstrates its good quality structure (**b**) Epitopes conserved in *A. baumannii* are shown on three dimensional structure.

Prediction of secondary structure revealed that BamA contains 184 amino acids in α-helices, 200 in β-sheets and 457 in random coils or loops (Suppl. Figure 2). STRING analysis showed that BamA interacts with other outer membrane protein assembly proteins present in *bam* operon such as BamB and BamD. BamA interacts with SurA also which is a chaperone in outer membrane. Other membrane associated proteins found to interact with BamA are LolE, a trans-membrane protein in lipoprotein releasing system, FabZ, a protein involved in unsaturated fatty acid synthesis and LptD, organic solvent tolerant protein (Suppl. Figure 3). The three dimensional structure of BamA was predicted by automated homology modelling to understand the structural features of BamA in *A. baumannii* and this structure contained a surface exposed β-barrel with several helices and loops, very similar to the structure determined in *E. coli* (Fig. 1a). B cell and T cell epitopes were predicted in BamA. As this protein is highly conserved among *A*. *baumannii* as well as other virulent species of *Acinetobacter*, therefore, MHC I and MHC II binding epitopes specific for more than 10 HLA alleles were predicted (Suppl. Table 2). Epitopes conserved in virulent species of *Acinetobacter* are shown on three dimensional structure of BamA that could be used to design broad spectrum vaccine^23^ (Fig. 1b).

### BamA is conserved in *Acinetobacter*

To explore the conservation of BamA, its sequence was subjected to BLASTp analysis that showed BamA is widely distributed among the 954 strains of Gram negative bacteria. It shared 92.3 to 99.9% sequence identity amongst the *A. baumannii* strains and 79.3% to 96.4% with other species of *Acinetobacter* (96.4% with *A. nosocomialis*, 95.4% with *A. genomosp. 33YU*, 93.7% with *A. pittii*, 93.4% with *A. seifertii*, 91.4% with *A. calcoaceticus*, 91.3% with *A. lactucae*, 91.2% with *A. oleivorans*, 83.9% with *A. haemolyticus*, 84.9% with *A. venetianus*, 83.6% with *A. junnii*, 84.9% with *A. beijerinckii*, 84.6% with *A. proteolyticus*, 84.6% with *A. gyllenbergii*, 82.3% with *A. venetianus*, 84.1% with *A. parvus*, 82.9% with *A. tjernbergiae*, 83% with *A. ursingii*, 80% with *A. radioresistens*, 79.9% with *A. indicus*, 79.4% with *A. soli* and 79.3% with *A. baylyi)*. BamA is widely present in *Pseudomonas aeruginosa* and *Klebsiella pneumoniae* strains but with lesser identity (38–40% and approximately 36% respectively). A phylogenetic tree based on the BamA sequences with significant identity is shown in Suppl. Figure 4. This widespread conservation of BamA demonstrates its broad spectrum vaccine potential against Gram negative bacteria. Further, colony PCR using *bamA* specific primers showed prevalence of *bamA* in 19 out of 20 clinical isolates including *A. baumannii* ATCC 19606 and ATCC 17978 (Suppl. Figure 5).

### BamA immunization elicited significant antibody titer

**Figure 2:**
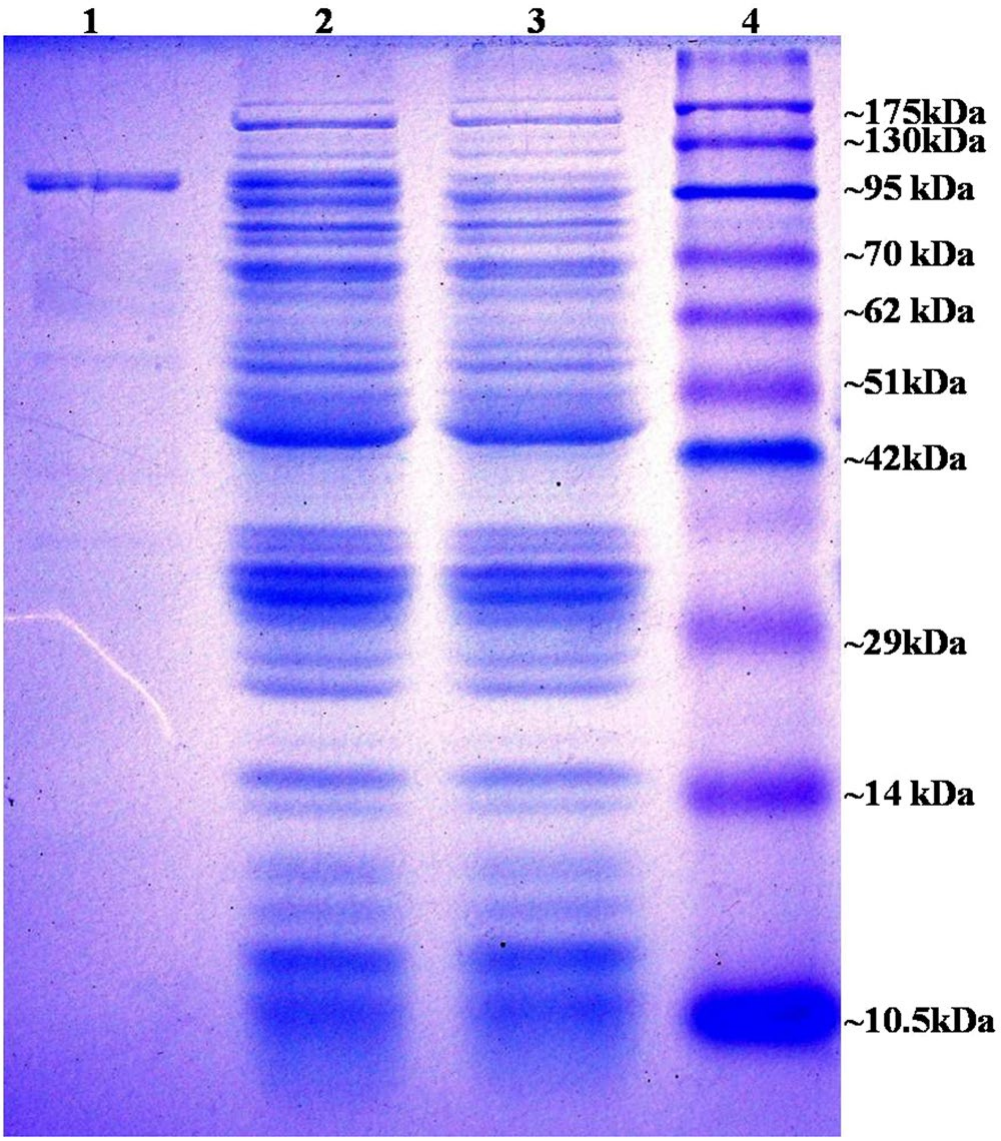
SDS PAGE (12%): Lane 1: purified BamA; lane 2, 3 induced and uninduced *E. coli* BL21 (pET28-a-*bamA*), lane 4: pink plus protein marker.

**Figure 3:**
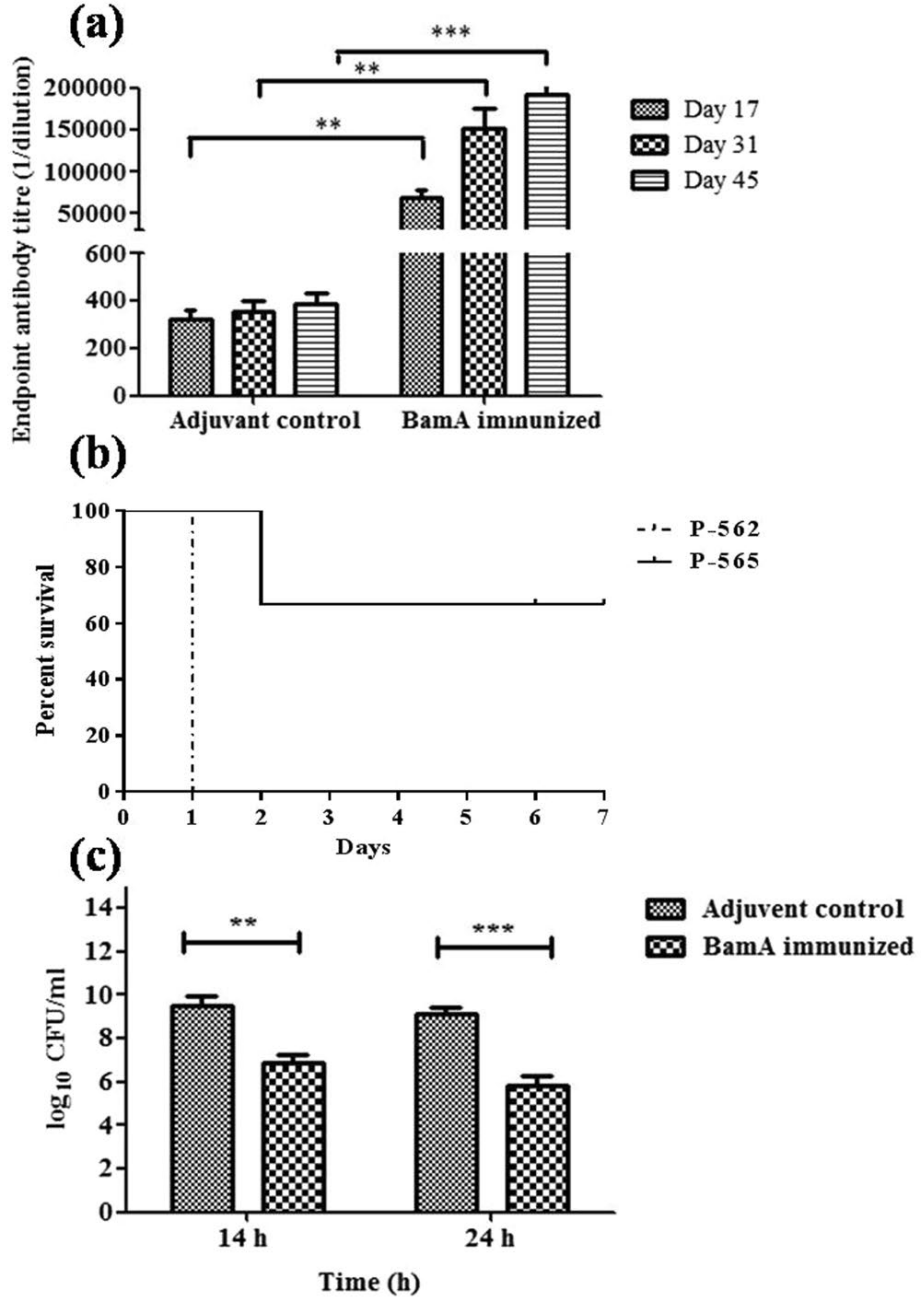
(**a**) Total IgG antibody levels in sera. Groups of female BALB/c mice (n = 10) were intraperitoneally immunized with 20 µg BamA formulated with 2% Al(OH)_3,_ at day 1, 14 and 28. Sera from adjuvant control and BamA immunized mice were collected 3 days after booster doses and on day 45 and IgG titer was determined. **p < 0.05, ***p < 0.001 (Adjuvant control vs BamA immunized mice). (**b**) MDR *A. baumannii* clinical isolates P562 and P565 (10^9^CFU/50 µl) were intranasally inoculated in mice (experiment was performed thrice with similar results -each time with n = 3). P562 showed 100% mice death within 24 h. (**c**) Bacterial burden in lungs. Groups of female BALB/c mice (n = 6) were immunized intraperitoneally with 20 µg BamA formulated with Al(OH)_3_ adjuvant on day 1, 14 and 28. The mice were intranasally challenged with 10^9^ CFU of *A. baumannii* clinical isolate P562 at day 45. Immunization with BamA reduced the bacterial burden by 3 log cycles in the lungs of pneumonia model mice sacrificed 14 (9.6 to 6.6 log cfu/ml) and 24 h (9 to 6 log cfu/ml) post infection. The data are presented as mean ± SD (experiment was performed thrice each time with n = 6). *p* value was determined by the one way analysis of variance (ANOVA). ***p < 0.001 (Adjuvant control vs immunized mice).

The purified renatured BamA (Fig. 2) showed < 1 EU/ml endotoxin level. Mice (n = 10) were immunized on day 1, 14 and 28 with 20 µg of purified BamA protein that generated antibody titer of >64000 after first, >128000 after second booster dose and 128000 to 256000 (mean value (n = 10) = 160000) at day 45 (Fig. 3a).

### Establishment of murine pneumonia model using virulent MDR *A. baumannii* clinical strain

I) Mice generally clear *A. baumannii* effectively when inoculated in less number^11,21^. Therefore, high doses were inoculated intraperitoneally in order to cause mice death so as to understand the effect of vaccination on mice survival. Out of five *A. baumannii* clinical strains, intraperitoneal injection of 10^7^ and 10^8^ CFU of two strains, P562 and P565, caused mice death within 48 h that showed their virulence.

II) To establish pneumonia model, these two strains were inoculated in mice intranasally to cause pneumonia. P-565 caused 66% mice death whereas P562 caused 100% mice death within 24 h at 10^9^ CFU dose (Fig. 3b). Therefore, P562 was selected for pneumonia model.

### BamA immunization reduced bacterial load in lungs

BamA immunization reduced bacterial burden in lungs by 3 log cycles each at 14 and 24 h as compared to unimmunized mice (Fig. 3c).

### BamA immunization reduced cytokines levels in lungs and serum

**Figure 4:**
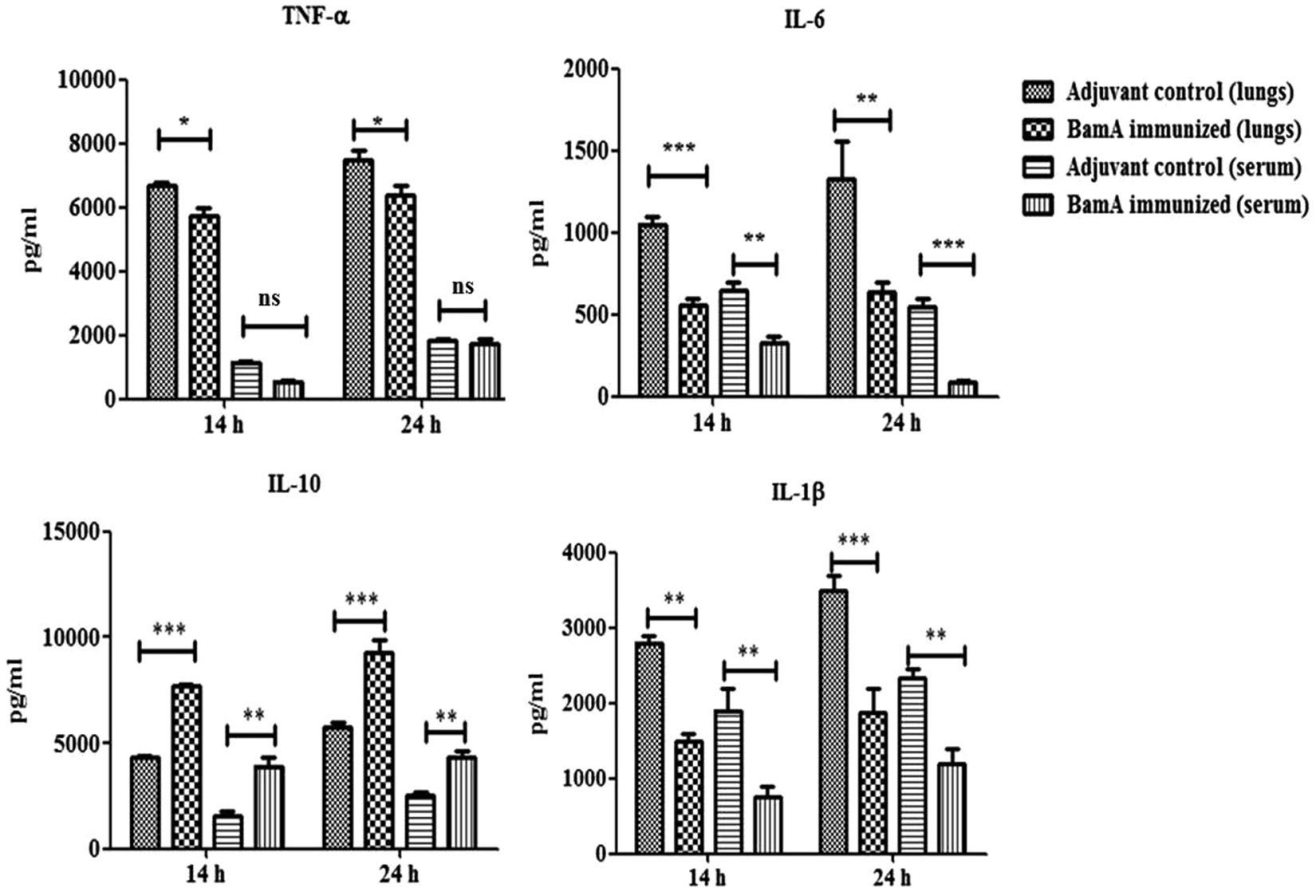
Cytokine levels in the lungs and sera. Groups of female BALB/c mice (n = 6) were immunized intraperitoneally with 20 µg BamA formulated with Al(OH)_3_ adjuvant on day 1, 14 and 28. The mice were intranasally challenged with 10^9^ CFU of *A. baumannii* clinical isolate P562 at day 45& cytokines levels determined 14 and 24 h post challenge. Detection limit of different cytokines was 1–10 pg/ml. *p* value was determined by the two way analysis of variance (ANOVA). *p < 0.1, **p < 0.05, ***p < 0.001, ns as non-significant (Adjuvant control vs BamA immunized mice). The data are presented as mean ± SD (experiment was performed thrice each time with n = 6).

Levels of pro- and anti-inflammatory cytokines in serum and lung tissue homogenate were determined to check whether BamA immunization was able to prevent the release of these cytokines (Fig. 4). Cytokine levels were notably high in tissue homogenate as compared to serum of mice due to bacterial inoculation in lungs by intra-nasal route and it did not cause septicemia. Levels of pro-inflammatory cytokines TNF-α (*p* < 0.05), IL-6 (*p* < 0.001) and IL-1β (*p* < 0.001)in tissue homogenate were significantly low in immunized mice at 14 h, and further reduced [TNF-α (*p* < 0.05), IL-6 (*p* < 0.05) and IL-1β (*p* < 0.001)] 24 h postinfection whereas anti-inflammatory cytokine IL-10 (*p* < 0.001) levels increased significantly as compared to unimmunized adjuvant control mice group. In case of serum cytokine analysis, there was reduction in the levels of pro-inflammatory cytokines IL-6 (*p* < 0.05) and IL-1β (*p* < 0.05) in immunized mice but TNF-α levels were comparable at 14 h. After 24 h, levels of pro-inflammatory cytokines IL-6 (*p* < 0.001) and IL-1β (*p* < 0.05) reduced significantly in immunized mice but TNF-α levels were comparable in immunized and unimmunized mice. Anti-inflammatory IL-10 levels increased significantly 14 h and 24 h postinfection (*p* < 0.05).

### Histological examination

**Figure 5:**
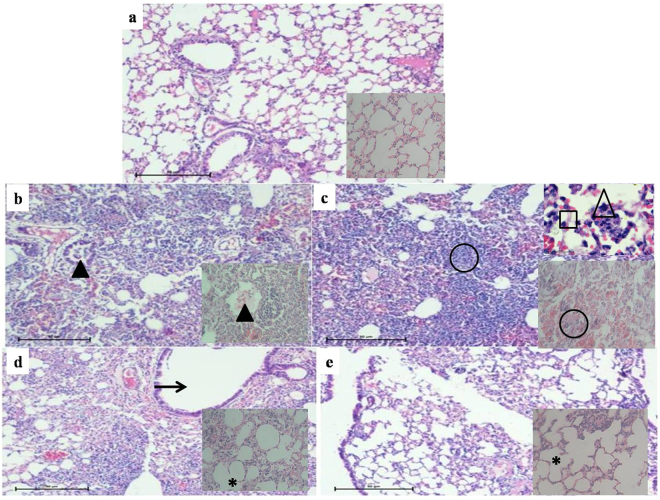
Lung histopathology (HE, 100X). Groups of female BALB/c mice (n = 10) were immunized intraperitoneally with 20 µg BamA formulated with Al(OH)_3_ adjuvant on day 1, 14 and 28. The mice were intranasally challenged with 10^9^ CFU of *A. baumannii* clinical isolate P562 at day 45.The mice were sacrificed 14 and 24 h post-challenge and lungs were collected for histopathology. (**a**) The lung from unimmunized uninfected mouse showed normal alveoli (inset 400X), (**b**) Unimmunized infected mice lungs 14 h postinfection showed peri- and endo-bronchial pneumonia packed with inflammatory cells (arrowhead), neutrohils in bronchus (inset 400X) (arrowhead), (**c**) Unimmunized infected mouse lung 24 h postinfection showed excessive consolidation packed with inflammatory cells in alveoli (circle, right bottom inset 400X); neutrophils (triangle) and bacterial colonies (square) are visible (right top inset 1000X) (**d**) Immunized infected mouse lung 14 postinfection showed clearance of inflammatory cells in bronchioles (arrow) and in alveoli (*) (inset 400X) and (**e**) Immunized infected mouse lung 24 h postinfection showed more clearance of inflammatory cells (*) (inset 400X). Bars = 50 µm.

Histological examination revealed that *A. baumannii* caused inflammation in the lungs known as pneumonia or consolidation. This is the solidification of alveoli by invasion of leucocytes. When a few areas are involved it is ‘focal pneumonia’. The bronchi in these lungs were found free and normal. In addition, there were congested blood vessels and free red cells in alveoli. In the infected mice, the leucocytes were containing *A. baumannii*. The extent of pneumonia was different at different time intervals such as at 14 h, infected unimmunized mice lungs were heavily consolidated with pathogen causing peri- and endo-bronchial pneumonia (score 3) which became severe at 24 h (score 4). On the other hand, immunized mice showed highly reduced inflammation and less infiltration of leucocytes in the alveoli at 14 h (score 2) that further reduced at 24 h (score 1) (Suppl. Figure 6a) (Fig. 5). Highest alveolar and vascular inflammation was observed in adjuvant control mice lungs 24 h postinfection (100%) as compared to mice lungs 14 h postinfection (90%). This inflammation reduced in immunized mice lungs 14 h (27.5%) and 24 h (7.34%) postinfection (Suppl. Figure 6b).

### Active and passive BamA immunization protects mice from lethal bacterial challenge

**Figure 6:**
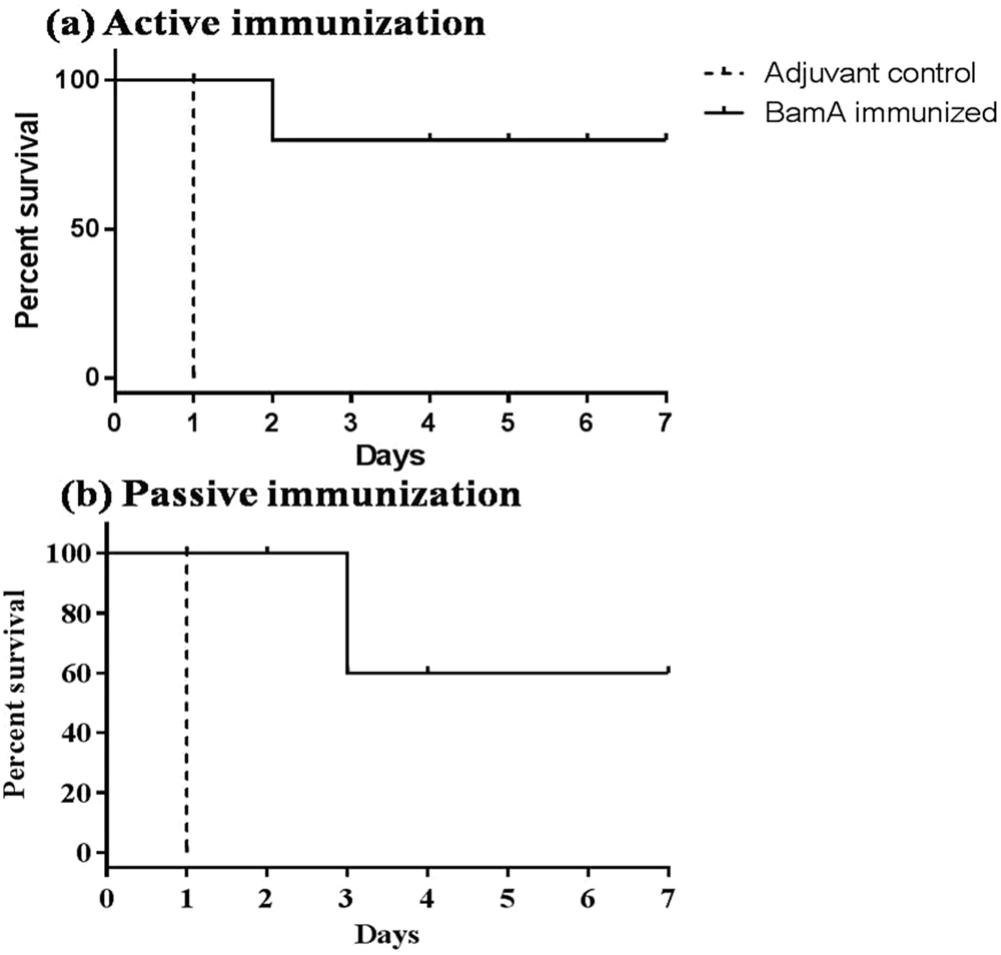
(**a**) Active immunization. Groups of female BALB/c mice (n = 10) were immunized intraperitoneally with 20 µg BamA formulated with Al(OH)_3_ adjuvant on day 1, 14 and 28. The mice were intranasally challenged with10^9^ CFU of *A. baumannii* clinical isolate P562 at day 45. BamA immunized mice showed 80% survival till seven days. (**b**) Passive immunization. Groups of female BALB/c mice (n = 10) were immunized intravenously with 100 µl BamA immunized mice sera. After 3 h, mice were intranasally challenged with 10^9^ CFU of *A. baumannii* clinical isolate P562. BamA passive immunization resulted in 60% survival as observed till seven days. Experiment was performed twice with similar results (n = 10 each time).

After determination of antibody titers at day 45, all of the mice in control and immunized group were challenged with lethal dose of *A. baumannii* to observe their mortality. BamA immunized (n = 10) mice showed highly improved survival rate as compared to unimmunized mice (n = 10) after challenge. All adjuvant control mice died within 24 h whereas BamA immunized mice showed 80% survival rate observed till seven days (Fig. 6a). For passive immunization, mice were injected with adjuvant control and BamA immunized mice sera 3 h prior to lethal bacterial challenge. All mice were observed for one week and 60% mice injected with immune sera survived as compared to adjuvant control sera injected mice that died (100%) within two days (Fig. 6b). This protection against lethal infection showed clearly that BamA possesses significant ability to evoke protective immune responses.

### Opsonophagocytic activity of antisera

**Figure 7:**
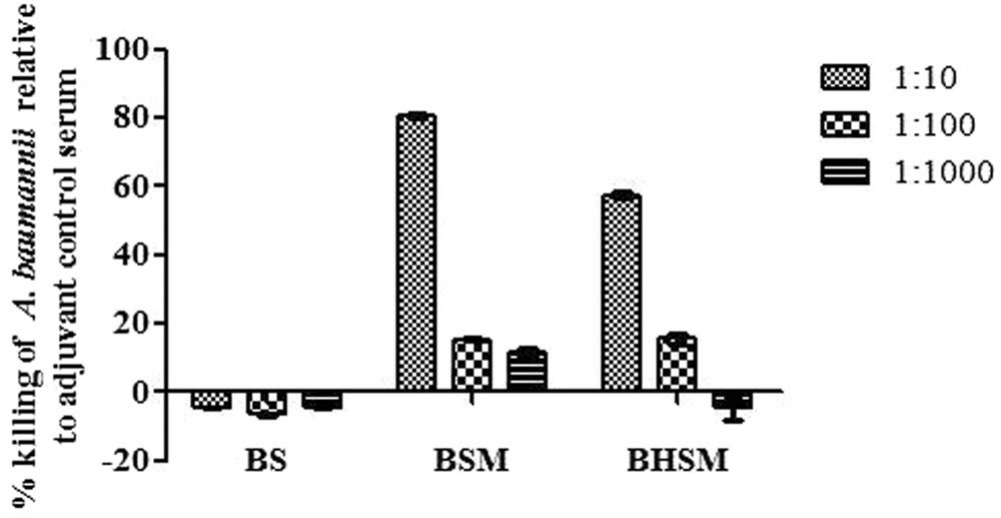
Antisera from BamA immunized mice showed effective opsonophagocytic killing of MDR *A. baumannii* clinical strain P-562. The experiment was performed by incubating bacteria (B) with serum (S), with serum and macrophages (BSM) and with heat inactivated serum along with macropahges (BHSM). The experiments were performed thrice and results were expressed as mean % killing of *A. baumannii* relative to adjuvant control serum.

To investigate the possible mechanism of mice protection from lethal bacterial challenge, the antisera obtained from the BamA immunized mice were checked for bacterial killing activity by opsonophagocytic assay (Fig. 7). After incubating bacteria with serum and macrophages, the killing was 81.8, 19.5 and 14.5% at serum dilutions of 1:10, 1:100 and 1:1000 respectively as compared to adjuvant control sera. Serum did not show any bactericidal activity without macrophages indicating that the bactericidal effects of serum were macrophage dependent. Heat inactivated serum showed the combinatorial opsonophagocytic activity of antibodies and bactericidal activity of complement components.

## Discussion

Reverse Vaccinology^24^ has emerged and established as a robust method for prediction and testing of vaccine candidate proteins against various pathogens, successful in case of *Neisseria meningitidis* ^25^, *Porphyromonas gingivalis* ^26^, *Chlamydia pneumoniae* ^27^, *Streptococcus pneumoniae* ^28^ and *Clostridium difficile* ^29^. It has changed the concept and approach to vaccine design and selection of novel antigens providing a route to investigate the mechanisms that underpin pathogenesis. RV has provided few potential sub-unit vaccine candidates for *A. baumannii* infections as well^17–19^. Recently, WHO has announced *A. baumannii* as the most critical MDR pathogen and vaccine development against it is a promising alternate treatment therapy as most of the commonly available antibiotics are not effective now^9^. Singh *et al*.^23^ screened the whole proteome of *A. baumannii* and selected potential vaccine candidates by Vaxign online tool on the basis of criteria required for an ideal vaccine candidate protein such as their sub-cellular localization, number of trans-membrane helices, adhesion probability, ability to bind to MHCs and similarity with human and mouse proteomes. This analysis resulted in a list of 51 potential vaccine candidates (Suppl. Table 4) out of which outer membrane β-barrel assembly protein, BamA was selected for investigation of its immunoprotective efficacy in murine pneumonia model established with a virulent MDR *A. baumannii* clinical isolate in this study. BamA is reported to be a part of Bam complex in *E. coli*, involved in the assembly and insertion of β-barrel proteins into the outer membrane^20^. Although BamA is not characterized in *A. baumannii* but it is highly conserved among the Gram negative bacteria due to its crucial role which makes it an ideal vaccine candidate protein to develop broad range vaccine against MDR pathogens. Its localization in outer membrane ensured its exposure to host immune system on encounter. There were no trans-membrane helices in BamA that signifies no part of protein is embedded and protein is completely available on outer membrane. Absence of trans-membrane helices also aids in cloning and expression of recombinant proteins. It showed high adhesion probability which helps in binding of pathogen to the host. As sub-unit vaccines are designed for broad range of protection by selecting only those proteins which are conserved throughout the strains of a specific species or species of a particular genus, conservation of BamA was investigated *in silico* using genomic and proteomic information available in databases and it was 92.3 to 99.9% conserved in different *A. baumannii* strains as well as shared significant similarity (78–94%) with other pathogenic species of *Acinetobacter*. Significant prevalence was found in MDR *A. baumannii* clinical isolates in this work that warrants its vaccine candidature. BamA contains numerous B cell as well as MHC I and MHC II binding epitopes specific for the HLA alleles prevalent in north Indian population^23^. Epitopes were predicted in different species of *Acinetobacter* and their docking with HLA-B*4405 allele (most prevalent in North India) and docking of Epitope- HLA-B*4405 complex to DM1-TCR illustrated its vaccine potential^23^. Prediction and validation of these antigenic peptides or epitopes could led to stitching them together to develop broad spectrum peptide vaccine against a group of Gram negative co-infecting pathogens such as *A. baumannii, P. aeruginosa, K. pneumoniae, E. coli* and *Burkholderia*. Further, BamA shares no similarity with human and mouse proteome which is absolutely essential to prevent autoimmune responses in the host.

The recombinant protein, when induced, accumulated in inclusion bodies. Therefore, this over-expressed protein was purified under denaturing conditions. Inclusion bodies were dissolved in various denaturing agents such as Guanidine hydrochloride, urea and SDS separately and solubility was found in the order: GnHCl > urea > SDS. Protein purification under denaturing conditions always provides maximum yield as compared to native expression^30,31^. Here, BamA purified under denaturing conditions provided significantly high yield of 65 mg/l. However, protein concentrations reduced drastically during refolding of denatured proteins to soluble form.

To monitor immunoprotective efficacy of BamA, an *A. baumannii* associated murine pneumonia model was established. *A. baumannii*, in most cases, causes pneumonia and respiratory tract is the most common site of infection and colonization^32,33^. *A. baumannii* ATCC 19606 reference strain has been widely used to develop pneumonia in mice models. Generally this strain and most clinical strains of *A. baumannii* do not cause infection in healthy mice and induce only a mild pneumonia that is self-limiting with low bacterial load in organs and low systemic dissemination. Therefore, to overcome these shortcomings, a virulent, MDR *A. baumannii* clinical strain was used to develop murine pneumonia model. Out of five MDR clinical isolates, the one, P-562, was most virulent and resistant to five antibiotics. Virulence was checked by intraperitoneal injection of different doses (10^5^–10^8^ CFU) of five MDR clinical isolates in mice and 10^7^ and 10^8^ CFU of two clinical isolates i.e. P-562 and P-565 caused mice death in 48 h (data not shown). Further, intranasal administration of higher doses (10^9^ CFU) of only P-562 caused mice death in 24 h. This infection model was used to investigate the immunoprotective efficacy of BamA so that the potential of sub-unit vaccine could be evaluated against currently prevailing MDR *A. baumannii* strains. Harris *et al*.^32^ reported that intraperitoneal injection of a hyper-virulent *A. baumannii* clinical isolate caused mice death within 24 h. Intratracheal bacterial administration has been reported by us to cause bacterial consolidation in lungs resulting in mice death within 24 h^18,19^. It was interesting to note in this work that when mice survive the bacterial challenge for at least 72 h, their survival rate increases to 100%. The possible reason could be that *A. baumannii* is quickly cleared from the mice lungs when administered in low count ( < 10^7^ CFU) and does not cause infection. Whereas in high doses (10^8^ or 10^9^ CFU), bacteria survive in enough number to release toxin compounds such as endotoxins and cause infection with serious damage to organs resulting in death.

A vaccine is considered effective when it protects the host from bacterial challenge. Although partial to complete protection in mice after lethal bacterial challenge has been reported^10–12,19,21,22,33^ by immunization with crude cell extract, outer membrane complex, outer membrane vesicles or recombinant proteins but the immunoprotective efficacy of subunit recombinant BamA is significant. Besides, BamA immunization reduced bacterial load in lungs by 3 log cycles, 14 h and 24 h postinfection as compared to adjuvant controls. Such considerable reduction is indicator of BamA efficacy. Inflammation, abscess, bronchitis and edema in the lungs are key identifiers of *A. baumannii* associated pneumonia. These markers along with oxidative stress result in death of lung epithelial cells^34^. BamA immunization resulted in decreased lung consolidation and infection, and reduced neutrophil infiltration in lungs.

Initiation of infection and inflammation in tissues is best studied by analyzing the pro- and anti-inflammatory cytokines in the serum or tissue homogenate. Levels of pro-inflammatory cytokines such as TNF-α, IL-6 and IL-1β are crucial indicators of cell death induced by *A. baumannii* ^10,21,22^. TNF-α is involved in systemic inflammation and initiates the caspase 3, 8 and 10 mediated apoptosis by binding to its specific receptor. It is mostly produced by macrophages, NK cells and neutrophils. Interleukin-6 plays dual role in modulating the expression of pro- or anti-apoptopic factors involved in the activation of intrinsic pathways of apoptosis^34^. It acts as both pro- and anti-inflammatory cytokine. In burns or tissue damage leading to swelling and inflammation, IL-6 is secreted by macrophages and T cells to stimulate the immune response. Changes in the levels of these cytokines in sera or tissue homogenate indicate the spread of infection. In this study, BamA immunization reduced the levels of pro-inflammatory cytokines (TNF-α, IL-6 and IL-1β) in sera as well as in lung tissue homogenate. Release of pro-inflammatory cytokines ensures the initiation of infection and reduction in their amounts signifies the effectiveness of vaccine. Elevation of anti-inflammatory cytokine IL-10 levels in sera and lungs in immunized mice indicated the recruitment of immune cells fighting with pathogen. As the route of bacterial inoculation was intranasal, levels of all the pro-inflammatory cytokines were significantly high in lung tissue homogenate as compared to sera due to bacterial consolidation in lungs.

The effectiveness of a vaccine is also indicated by the antibody response generated by the host. BamA immunization evoked a significant humoral response (IgG antibody titer > 128000). These anti-BamA antibodies seem to control the infection by binding to *A. baumannii* surface and promoting immune system activities such as opsonisation, phagocytosis and killing of the pathogen by natural killer cells, macrophages and neutrophils. Moreover, these antibodies along with macrophages also activated complement system which led to lysis of *A. baumannii* and also induced robust localized production of immune cells that generated an effective inflammatory response. Once bacterial count decreases in organs by antibody mediated neutrilization, mice survival increases significantly as seen in case of immunization with OMCs^12^, OmpW^21^, Omp 22^22^ and NucAb^19^. Therefore, the possible mechanism for bacterial clearance after BamA immunization was investigated by *in vitro* opsonophagocysis assays. Reduction in bacterial count when incubated with antisera and macrophages showed a combinatorial antibody-macrophage killing action. However, without macrophages, no bacterial killing demonstrated that antibodies were macrophage dependent where antibodies opsonize and macrophages kill the pathogen.

BamA passive immunization resulted in 60% protection from lethal bacterial dose. Direct administration of specific antibodies by passive immunization has been reported for improved survival due to bacterial neutralization and antibody or complement-mediated opsonophagocytic killing^19,22^. BamA is the key protein in outer membrane assembly complex and assembly of proteins in outer membrane might be hampered due to binding of immune complexes to BamA resulting in cell death due to lack of essential proteins in the membrane.

In this study, for the first time, *A. baumannii* outer membrane assembly protein BamA is reported to show significant immunoprotective efficacy. BamA immunization produced significant titer of macrophage dependent opsonophagocytic antibodies and conferred 80% immunoprotection against lethal bacterial challenge by reducing bacterial load in lungs and pro-inflammatory cytokine levels in sera and tissue homogenate. Compared to the earlier vaccine development efforts,^17,19,21–23^ BamA is a promising vaccine candidate against infections caused by *A. baumannii*.

## Materials and Methods

### Animals, ethical clearance and bacterial strains

Animal studies were approved by the Animal Ethics Committee of Panjab University. 6–8 weeks old pathogen-free female Balb/c mice were housed in clean polypropylene cages and fed a standard antibiotic-free diet (Hindustan Lever Products, Kolkata, India) and water *ad libitum*. All experiments were performed in accordance with the guidelines of Committee for the Purpose of Control and Supervision of Experiments on Animals (CPCSEA), Government of India. *A. baumannii* ATCC 19606 was procured from ATCC, MDR *A. baumannii* clinical isolates were obtained from Government Medical College and Hospital, Chandigarh and a highly virulent MDR strain P-562 (resistant to Amikacin, Tobramycin, Ceftriaxone, Ampicillin/Sulbactum and Imipenem) was used to establish murine pneumonia model (Suppl. Table 3). *E.coli* BL21 (DE3) and pET28-a plasmid from Novagen were used for cloning and expression of BamA. The bacterial strains were grown in Luria-broth (LB) containing kanamycin (25 µg/ml), wherever required.

### *In silico* analysis of *A. baumannii* ATCC 19606 proteome

BamA was predicted as potential vaccine candidate by Vaxign (Suppl. Table 4)^35^. The possible domains and characteristic motifs contained in BamA were investigated by BLAST (http://blast.ncbi.nlm.nih.gov/Blast.cgi) and InterPRO v46.0 (http://www.ebi.ac.uk/inter-pro/). Physicochemical analysis including molecular weight, theoretical pI, instability index, aliphatic index, amino acid composition, and grand average of hydropathicity (GRAVY) was performed using ProtParam (http://web.expasy.org/protparam/). The secondary structure elements were determined using GOR IV (https://npsa-prabi.ibcp.fr/NPSA/npsa_gor4.html) and SOPMA (https://npsa-prabi.ibcp.fr/NPSA/npsa_sopma.html). SignalP 4.1 online tool was used to predict the signal sequence and the signal cleavage site (http://www.cbs.dtu.dk/services/SignalP/). Tertiary structure of BamA was predicted by SWISS-MODEL.

### Cloning, expression and purification of BamA

Chromosomal DNA of *A. baumannii* ATCC 19606 was isolated^36^ and used as template for PCR. Primers were designed by online tool ‘OligoEvaluator™’ for BamA amplification having BamHI and XhoI restriction sites in forward (5′- ATA**GGATCC**TGTGGTGGAGGAAGTT-3′) and reverse (5′- TCA**CTCGAG**TTATTTTGTCTTAATTTGATAACAAT-3) primers respectively. PCR reaction was performed with initial denaturation at 94 °C for 3 min followed by 33 thermal cycles of denaturation at 95 °C for 1 min, annealing at 58 °C for 30 sec, and extension at 72 °C for 3 min. Final extension was carried out at 72 °C for 10 min. The BamHI and XhoI digested PCR product was ligated to similarly digested pET-28a and transformed into *E. coli* BL21 (DE3) by electroporation. Transformants were selected on LB-kanamycin agar plates and confirmed by PCR.

For protein production, a single colony of recombinant *E. coli* BL21 (DE3) strain was inoculated into 10 ml LB containing kanamycin and incubated overnight at 37 °C. The 1.5 ml of culture was used to inoculate 250 ml LB containing kanamycin and incubated at 37 °C/180 rpm until OD_600_ reached 0.6. 1 mM IPTG was added and the culture was incubated for 5 h at 37 °C, centrifuged, pellet was suspended in 25 ml of buffer (100 mM phosphate buffer, 300 mM NaCl, pH 8), sonicated and dissolved in urea lysis buffer (8 M Urea, 100 mM phosphate buffer, 300 mM NaCl, pH 8) and purified by Ni-NTA chromatography.

The protein was refolded by urea gradient dialysis method according to Qiagen’s guidelines. Briefly, the protein was dialyzed against refolding buffer (300 mM NaCl, 100 mM phosphate buffer, 0.1 M arginine, pH 8) containing 6 M urea for 2 h. Urea concentration in refolding buffer was decreased to 4 M and dialyzed for 2 h. Then, urea concentration was decreased to 1 M and dialyzed for 2 h. Finally, protein was dialyzed against PBS overnight at 4 °C. Renatured BamA was used for mice immunization.

Protein concentration was estimated by Bradford protein estimation kit (Bangalore Genei India Pvt. Ltd.). Pyrogenicity of proteins was checked by cartridge based PTS (point-of-use, portable test system, Charlesriver, USA) which is based on principle of kinetic chromogenic technique. The *Limulus* amebocyte lysate (LAL) reagent upon interacting with the endotoxins present in the samples results in initiation of cascade of reactions and formation of colored substrate. Sample (25 µL) was added to each well in the PTS cassette and was incubated at 38 °C for 15–20 min for the reaction to take place. All observations were noted with 50–200% spike recovery. The test was sensitive up to 0.01 Endotoxin Unit (EU)/mL.

### Establishment of MDR ***A. baumannii*** associated murine pneumonia model

I) To check the virulence of five MDR *A. baumannii* clinical strains, 10^5^, 10^6^, 10^7^ and 10^8^ CFU were injected intraperitoneally in mice groups (n = 3 for each dose) and their survival was observed for seven days. Control group (n = 3) was administered with normal saline.

II) Then, to establish *A. baumannii* associated pneumonia, the strains were administered intranasally (10^7^, 10^8^, 10^9^ CFU of each) in mice (n = 3 for each dose) and their survival was noted for seven days. (Control n = 3).

### Evaluation of immunoprotective efficacy of BamA in mouse model

#### Mouse immunization

BamA specific antibodies were raised in mice by injecting purified protein with adjuvant.

Refolded BamA protein (0.4 mg/ml) was mixed with 2% Al(OH)_3_ (Sigma) in 1:1 ratio and a group of mice (n = 6) was administered 100 µl of protein-adjuvant mixture intraperitoneally. Control group (n = 6) was injected with adjuvant only. Booster doses of BamA (20 µg) were given with Al(OH)_3_ at 14^th^ and 28^th^ day and sera of control and immunized mice were collected at day 17, 31 and 45.

#### Antibody titer

BamA specific IgG antibodies were measured by ELISA^18^. Briefly, 200 ng BamA in sodium bicarbonate buffer (pH 9.6) was coated to each well of 96 well microtire plate (NUNC) by incubation at 4 °C overnight. The wells were washed thrice with 0.1% Tween 20 in PBS (PBST) and blocked with 5% BSA in PBST (PBSTM) for 1 h at room temperature. Sera were serially diluted two fold in PBSTM and added to wells followed by incubation for 1 h at 37 °C. Wells were washed thrice with PBST and 100 µl of horseradish peroxidase-conjugated anti-IgG (Bangalore Genei) diluted in PBSM (1:5,000) was added to each well and incubated at room temperature for 1 h. Wells were again washed with PBST thrice and 100 µl of 3,3′,5,5′-Tetramethylbenzidine or TMB (Sigma) was added to each well and developed for 20 min at room temperature. The reaction was stopped with the addition of 100 µl of 2 M HCl, and the absorbance was read at 450 nm. The endpoint titer was defined as the highest dilution at which the optical density was 0.1 greater than that of control wells receiving control adjuvant serum.

#### Cytokines levels estimation

Lung tissue homogenate and sera were collected from adjuvant control and BamA immunized mice 14 and 24 h post infection. 24 h samples from adjuvant control mice were collected as soon as the mice died. Levels of cytokines TNF-α, IL-6, IL-1β and IL-10 were estimated using GenAsia, Philippines kits according to the supplier’s instructions. Concentrations were calculated in Graphpad Prism 5 software.

#### Determination of bacterial load in lungs

Mice were sacrificed by cervical dislocation and dissected aseptically to remove lungs. The lungs were suspended in 1 ml PBS and homogenized. Homogenates were serially diluted, spread plated on LB agar and incubated at 37 °C overnight. The number of colony forming units was counted and the results were expressed as log CFU.

#### Opsonophagocytic killing assay

Balb/c murine macrophage cells were cultured in RPMI medium with 5% fetal calf serum (FCS) and harvested by scraping with cell scrapers (ThermoScientific). The macrophages (3.2 × 10^6^ cells/ml), *A. baumannii* (2.2 × 10^6^ CFU/ml) and BamA immunized or adjuvant control sera (dilution 1:10, 1:100 and 1:1000) were mixed and incubated for 1 h at 37◦C. A subset of sera was subjected to heat treatment at 56◦C for 30 min to inactivate the endogenous complement components. Finally, the mixtures were diluted and plated for bacterial counting^21^.

#### Histological examination

Aseptically collected lung specimens were fixed in 10% buffered formalin, stained with hematoxylin-eosin and observed under microscope at 40, 100 and 400X magnification. Histological examination primarily included the assessment of cellular infiltration and aggregation by scoring the number of immune cells (neutrophils) at a magnification of × 400. When significant infiltration was seen in the same specimen, the mean for several areas was determined and the specimen was scored accordingly^37^. They were classified as absent (score of 0) when there were no or fewer than 69 cells per high-power field (HPF) (at a magnification of × 400), mild (score of 1) for 70 to 149 cells per HPF, moderate (score of 2) for 149 to 249 cells per HPF, severe (score of 3) for 250 to 499 and very severe (score 4) for 500 or more per HPF. These histopathological scores, evaluating the alveolar and vascular inflammation, were used to calculate percentages of total alterations.

### Active and passive immunization for survival against challenge with lethal dose of *A. baumannii*

Group of mice (n = 10) was immunized intraperitoneally with 20 µg BamA formulated with 2% Al(OH)_3_ adjuvant on day 1, 14 and 28, and intranasally challenged with 10^9^ CFU of *A. baumannii* clinical strain P-562 at day 45. The survival rate of mice was recorded continuously over the next seven days. For passive immunization, 100 µl adjuvant control sera and immunized mice sera were administered in mice groups (n = 10) via tail vein intravenous injection. After 3 h, mice were challenged intranasally with optimized lethal dose (10^9^ CFU) of *A. baumannii* clinical isolate P-562. The survival rate of mice was recorded continuously over the next seven days

### Statistical Analyses

All statistical analyses were performed using Graphpad Prism5 software. The data was presented as mean with standard deviations represented as error bars. Student t-test and one/two way analysis of variance (ANOVA) were applied for all the comparisons. Survival rates were analyzed by log-rank test. Results were considered significant at *p* < 0.05.

## Electronic supplementary material

Suppl. data
