# Supplementary material for: Immunoprotective potential of BamA, the outer membrane protein assembly factor, against MDR Acinetobacter baumannii
Source: Sci Rep. 2017 Sep 29;7:12411. doi: 10.1038/s41598-017-12789-3 (PMC5622086; doi:10.1038/s41598-017-12789-3)
Supplement: Supplementary file 1 — Suppl. data [file 41598_2017_12789_MOESM1_ESM.pdf]

Supplementary data:

Immunoprotective potential of BamA, the outer membrane protein assembly factor, against MDR *Acinetobacter baumannii*

Ravinder Singh[1], Neena Capalash[2], Prince Sharma[1*]

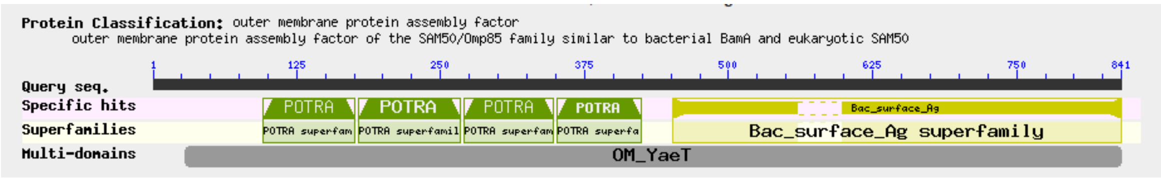

Suppl. Fig. 1: BLASTp was performed to locate domains available in BamA sequence

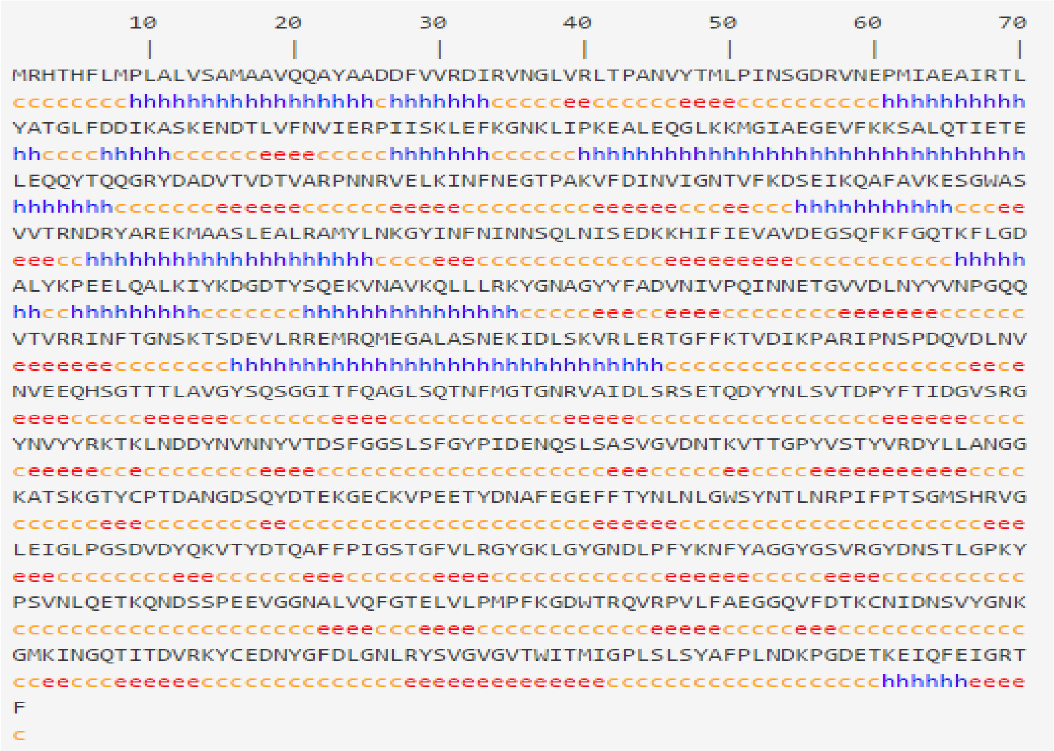

GOR4 :				
Alpha helix	(Hh)	:	184 is	21.88%
3_{10} helix	(Gg)	:	0 is	0.00%
Pi helix	(Ii)	:	0 is	0.00%
Beta bridge	(Bb)	:	0 is	0.00%
Extended strand	(Ee)	:	200 is	23.78%
Beta turn	(Tt)	:	0 is	0.00%
Bend region	(Ss)	:	0 is	0.00%
Random coil	(Cc)	:	457 is	54.34%
Ambiguous states	(?)	:	0 is	0.00%
Other states		:	0 is	0.00%

Suppl. Fig.2: Secondary structure prediction by Phyre2 and GOR IV showing amino acid wise secondary structures of BamA: alpha helix- 184 amino acids, extended strand- 200 amino acids and random coil- 457 amino acids

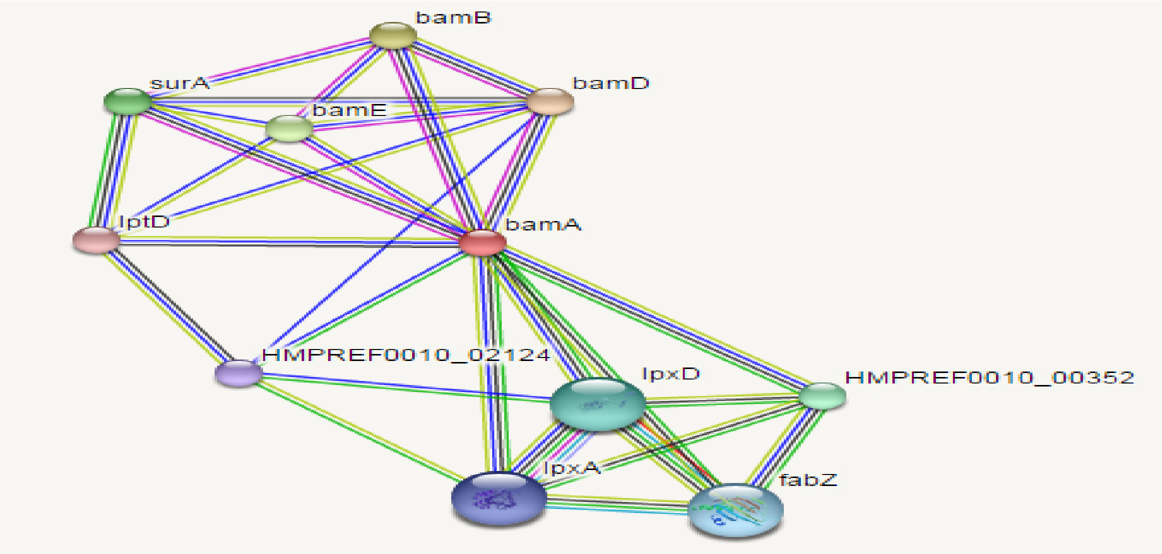

Your Input:

bamA	*Outer membrane protein assembly factor BamA; Part of the outer membrane protein assembly complex, which is involved in assembly and insertion of beta-barrel proteins into the outer membrane (By similarity) (841 aa)*

Predicted Functional Partners:

		Neighborhood	Gene Fusion	Cooccurence	Coexpression	Experiments	Databases	Textmining	[Homology]	Score
bamD	*Outer membrane protein assembly factor BamD; Part of the outer membrane protein assembly complex, which is...*			●	●	●		●		0.999
bamB	*Outer membrane protein assembly factor BamB; Part of the outer membrane protein assembly complex, which is...*			●	●	●		●		0.999
bamE	*Outer membrane protein assembly factor BamE; Part of the outer membrane protein assembly complex, which is...*			●		●		●		0.996
surA	*Rotamase SurA; Chaperone involved in the correct folding and assembly of outer membrane proteins. Recognize...*			●	●	●		●		0.996
HMPREF0010_00352	*RIP metalloprotease RseP (451 aa)*	●		●	●			●		0.973
lpxD	*UDP-3-O-acylglucosamine N-acyltransferase; Catalyzes the N-acylation of UDP-3-O-acylglucosamine using 3-hydr...*	●		●	●			●		0.965
fabZ	*Beta-hydroxyacyl-ACP dehydratase; Involved in unsaturated fatty acids biosynthesis. Catalyzes the dehydration o...*	●			●			●		0.955
lpxA	*Acyl-[acyl-carrier-protein]--UDP-N-acetylglucosamine O-acyltransferase; Involved in the biosynthesis of lipid A, a p...*	●		●	●			●		0.945
HMPREF0010_02124	*Lipoprotein releasing system, transmembrane protein LolE (411 aa)*	●		●						0.923
lptD	*Organic solvent tolerance protein; Involved in the assembly of LPS in the outer leaflet of the outer membrane. Det...*			●	●			●		0.906

Suppl. Fig. 3: Graphic display of BamA interacting proteins by STRING analysis

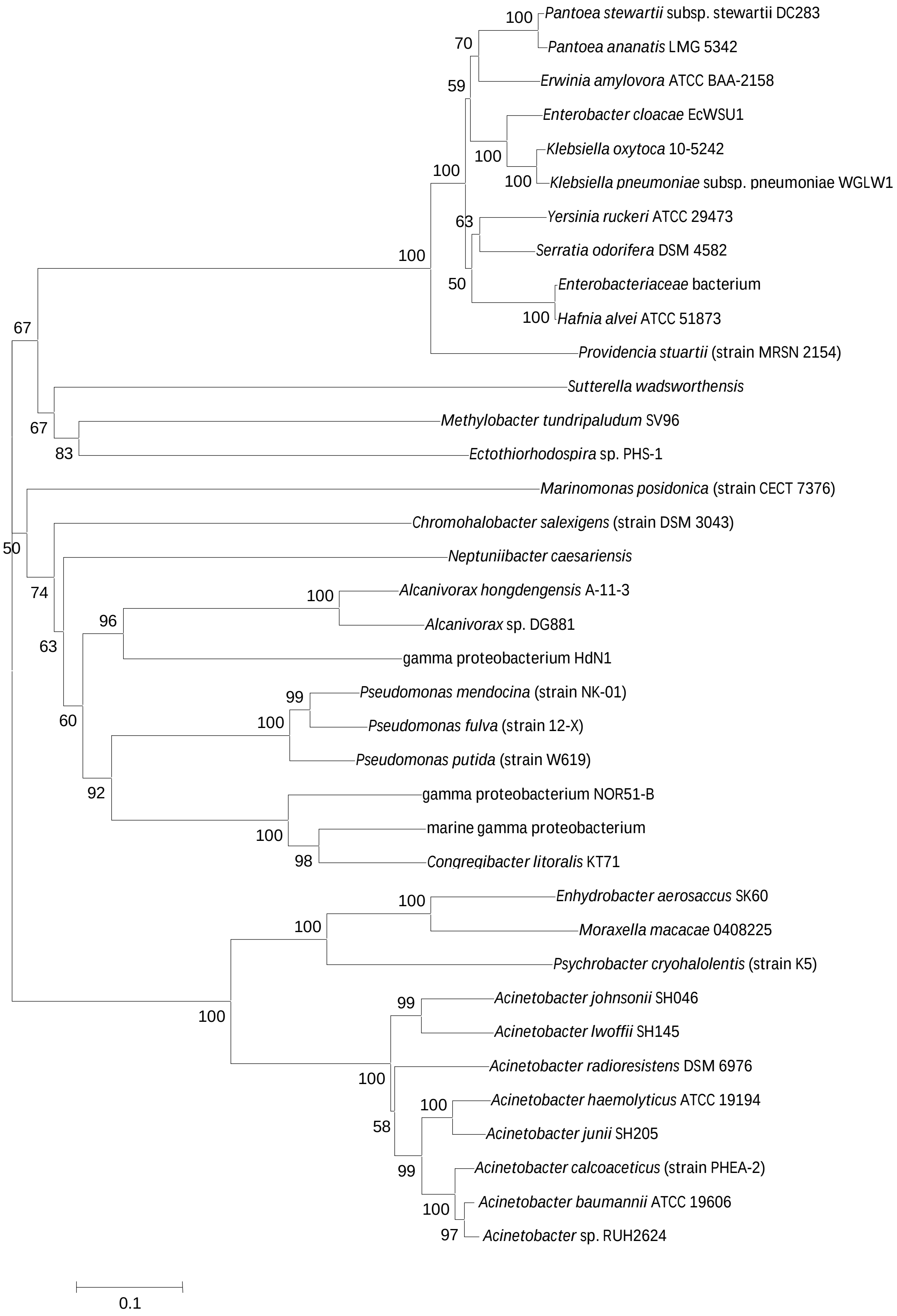

100
Pantoea stewartii subsp. stewartii DC283
Pantoea ananatis LMG 5342
70
Erwinia amylovora ATCC BAA-2158
59
Enterobacter cloacae EcWSU1
100
Klebsiella oxytoca 10-5242
100
Klebsiella pneumoniae subsp. pneumoniae WGLW1
100
63
Yersinia ruckeri ATCC 29473
Serratia odorifera DSM 4582
100
50
Enterobacteriaceae bacterium
100
Hafnia alvei ATCC 51873
Providencia stuartii (strain MRSN 2154)
67
Sutterella wadsworthensis
67
Methylobacter tundripaludum SV96
83
Ectothiorhodospira sp. PHS-1
Marinomonas posidonica (strain CECT 7376)
Chromohalobacter salexigens (strain DSM 3043)
50
Neptuniibacter caesariensis
74
100
Alcanivorax hongdengensis A-11-3
96
Alcanivorax sp. DG881
63
gamma proteobacterium HdN1
99
Pseudomonas mendocina (strain NK-01)
100
Pseudomonas fulva (strain 12-X)
60
Pseudomonas putida (strain W619)
92
gamma proteobacterium NOR51-B
marine gamma proteobacterium
100
98
Congregibacter litoralis KT71
100
Enhydrobacter aerosaccus SK60
100
Moraxella macacae 0408225
Psychrobacter cryohalolentis (strain K5)
99
Acinetobacter johnsonii SH046
100
Acinetobacter lwoffii SH145
Acinetobacter radioresistens DSM 6976
100
100
Acinetobacter haemolyticus ATCC 19194
58
Acinetobacter junii SH205
99
Acinetobacter calcoaceticus (strain PHEA-2)
100
Acinetobacter baumannii ATCC 19606
97
Acinetobacter sp. RUH2624
0.1

Suppl. Fig. 4: A phylogenetic tree based on BamA sequences present in various organisms was constructed using MEGA software, version 4.0, based on maximum likelihood.

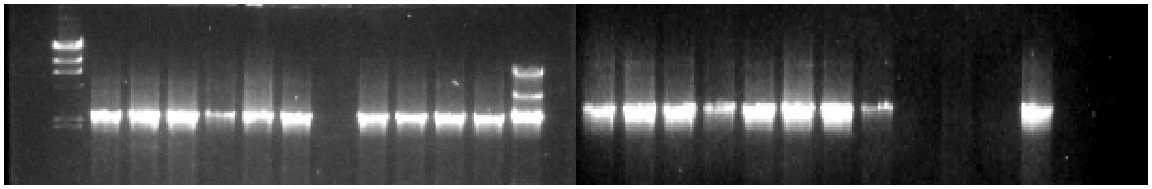

Suppl. Fig. 5: ***bam A*** **prevalence was checked in clinical isolates by colony PCR.** ***bamA*** **was present in 19/20** ***A. baumannii*** **clinical isolates**

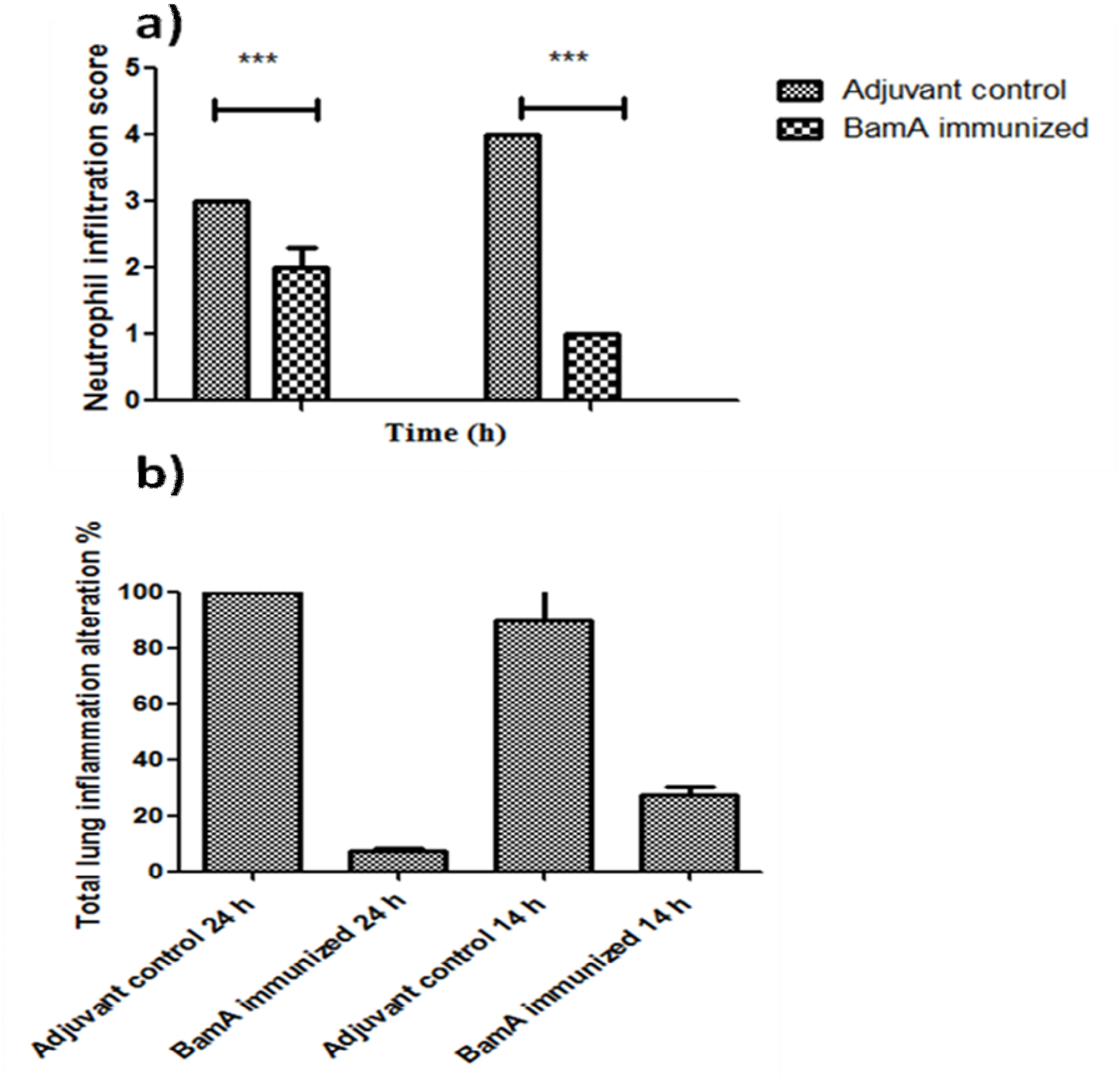

Suppl. Fig. 6: a) Fig. 5: Lung histopathology (HE, 100X). Groups of female BALB/c mice (n=10) were immunized intraperitoneally with 20 µg BamA formulated with $Al(OH)_3$ adjuvant on day 1, 14 and 28. The mice were intranasally challenged with 10^9 CFU of *A. baumannii* clinical isolate P562 at day 45.The mice were sacrificed 12 and 24 h post-challenge and lungs were collected for histopathology. a) Neutrophil infiltration score (B) Total inflammation alteration %age scored in tissue section of murine lungs stained with H&E. The data are pooled from at least two independent experiments (n = 6). Values represent the mean standard error of the mean(SEM) and compared with adjuvant control 24 h (100%). ***p < 0.001.

Suppl. Table 1: Physico-chemical parameters of BamA

S. No.	Parameter	BamA
1.	Gene ID	DOC6H3
2.	Number of aminoacids	841
3.	Molecular weight	93.78 kDa
4.	Therotical pI	5.21
5.	Total number of negatively charged residues (Asp + Glu)	103
6.	Total number of positively charged residues (Arg + Lys)	88
7.	Sub-cellular localization	Outer membrane
8.	Signal peptide	1-24 aminoacids
9.	Adhesion probability	0.539
10.	Number of trans-membrane helices	0
11.	Similarity with human, mouse and pig proteome	No
12.	Grand average of hydropathicity (GRAVY)	-0.447
13.	Extinction coefficient	$93770M^{-1}cm^{-1}$

Suppl.Table 2: B cell epitopes predicted by IEDB B cell prediction tool and and T cell epitopes of BamA predicted by ProPred and ProPred I.

B cell epitopes with score more than 0.85 are mentioned. All the highest scorer CTL and HTL epitope sequences were selected at a threshold of 4% for ProPred 1 with proteasome filter at 5% & 1% for ProPred. Only epitopes binding to more than 10 MHC alleles are shown.

IEDB			Propred			Propred I		
**Amino acid position**	**B cell epitopes sequence**	**score**	**Amino acid position**	**Predicted CTL epitopes sequence**	**Number of MHC class II binding alleles**	**Amino acid position**	**Predicted HTL epitopes sequence**	**Number of MHC class I binding alleles**
261	EVAVDEGSQFKFGQTK	0.94	7	FLMPLALVS	23	15	AMAAVQQAY	15
435	GYSQSGGITFQAGLSQ	0.93	13	LVSAMAAVQ	30	32	DIRVNGLVR	12
759	KCNIDNSVYGNKGMKI	0.92	30	VRDIRVNGL	17	49	MLPINSGDR	11
574	NGDSQYDTEKGECKVP	0.91	31	LVRLTPANV	49	67	IRTLYATGL	12
563	TSKGTYCPTDANGDSQ	0.91	46	VYTMLPINS	38	100	KLEFKGNKL	15
498	TKLNDDYNVNNYVTDS	0.91	67	IRTLYATGL	14	133	ALQTIETEL	12
486	GVSRGYNVYYRKTKLN	0.9	90	FNVIERPII	15	222	KMAASLEAL	14
148	QGRYDADVTVDTVARP	0.9	108	LIPKEALEQ	19	226	LEALRAMYL	11
826	PGDETKEIQFEIGRTF	0.89	118	LKKMGIAEG	17	280	DALYKPEEL	14

747	VLFAEGGQVFDTKCNI	0.89	230	LRAMYLNKG	23	434	VGYSQSGGI	11
464	LSRSETQDYYNLSVTD	0.88	239	YINFNINNS	33	479	DPYFTIDGV	13
22	AYAADDFVVRDIRVNG	0.88	313	LRKYGNAGY	13	522	YPIDENQSL	19
94	ERPIISKLEFKGNKLI	0.87	373	MRQMEGALA	24	600	EFFTYNLNT	11
76	FDDIKASKENDTLVFN	0.87	404	IKPARIPNS	24	607	NLGWSYNTL	11
683	GGYGSVRGYDNSTLGP	0.86	494	YYRKTKLND	16	652	FPIGSTGFV	14
313	LRKYGNAGYYFADVNI	0.86	546	YVSTYVRDY	23	697	GPKYPSVNL	13
270	FKFGQTKFLGDALYKP	0.86	550	YVRDYLLAN	18	722	ALVQFGTEL	16
692	DNSTLGPKYPSVNLQE	0.85	662	LRGYGKLGY	37	754	QVFDTKCNI	11
543	TGPYVSTYVRDYLLAN	0.85	723	LVQFGTELV	19	814	LSLSYAFPL	16
294	YKDGDTYSQEKVNAVK	0.85	808	ITMIGPLSL	21	833	IQFEIGRTF	12

Supp. Table 3: *A. baumannii* clinical isolates and their antibiotic sensitivity

**S. no.**	**Name**	**Source**	**Resistant to**	**Sensitive to**
1.	P-562	Tracheal (ICU)	AMK, TOB, CRO, SAM, IPM	CST/PMX
2.	P-563	Tracheal (ICU)	AMK, CRO, SAM, IPM	TOB, CST/PMX
3.	P-565	Tracheal (ICU)	AMK, TOB, CRO, SAM, IPM	CST/PMX
4.	P-568	Tracheal (ICU)	AMK, CRO, SAM, IPM	TOB, CST/PMX
5.	P-569	Tracheal (ICU)	AMK, TOB, CRO, SAM, IPM	CST/PMX

AMK, Amikacin; TOB, Tobramycin; CRO, Ceftriaxone; SAM, Ampicillin/Sulbactum; IPM, Imipenem; CST, Colistin; PMX, Polymyxin B.

Suppl. Table 4: Vaccine candidate proteins predicted by Vaxign fulfilling the criteria of ideal vaccine candidate localized in outer membrane, with adhesion probability greater than 0.5 and less than 2 trans-membrane helices.

S. No.	UniProt ID	Protein Note	Outer membrane probability	Adhesin Probability	Trans-membrane helices	Protein Length
1	D0CBN6	Carbapenem-associated resistance protein	0.952	0.668	0	249
2	D0CDB7	TonB-dependent vitamin B12 receptor	0.993	0.649	0	616
3	D0CDV8	Outer membrane receptor protein	0.952	0.502	0	772
4	D0C9R5	Type VI secretion system OmpA/MotB	1	0.606	0	217
5	D0CDG4	Outer membrane receptor protein	1	0.519	0	723
6	D0CC45	TonB-dependent receptor	1	0.557	0	924
7	D0CDE3	Porin B	0.993	0.654	1	417
8	D0CE63	Porin	1	0.525	0	414
9	D0CF71	Outer membrane protein	0.952	0.554	0	299
10	D0CAH3	Outer membrane receptor for monomeric catechols	1	0.612	0	743
11	D0C7X6	Outer membrane usher protein mrkC	1	0.631	0	809
12	D0C682	Outer membrane receptor protein	1	0.547	0	745
13	D0C7J7	Ferric aerobactin receptor	1	0.597	0	772
14	D0CFN5	Predicted protein	0.949	0.752	0	209
15	D0CC21	Ferric anguibactin receptor	0.995	0.629	0	766
16	D0C9M4	Competence lipoprotein comL	0.992	0.509	0	385
17	D0CFK2	TonB-dependent receptor protein	1	0.593	0	736
18	D0C803	Putative uncharacterized protein	0.952	0.651	0	443
19	D0C6S0	Porin B	1	0.613	0	439
20	D0C7C2	Outer membrane receptor protein	1	0.628	0	735
21	D0C771	Putative uncharacterized protein	0.949	0.572	0	324
22	D0CB83	Putative uncharacterized protein	0.952	0.645	0	270
23	D0C6Y7	FhaB protein	0.995	0.671	0	1072

S. No.	UniProt ID	Protein Note	Outer membrane probability	Adhesin Probability	Trans-membrane helices	Protein Length
24	D0CDL7	Outer membrane transporter	0.952	0.635	0	476
25	D0CDY8	Outer membrane receptor protein	1	0.505	0	704
26	D0CF50	Porin	0.952	0.599	0	255
27	D0C998	Copper resistance protein B	0.993	0.549	1	272
28	D0C689	TonB-dependent siderophore receptor	1	0.591	0	718
29	D0C7P2	Outer membrane receptor protein	1	0	0	862
30	D0C9N1	Putative uncharacterized protein	0.952	0.571	0	394
31	D0C8G8	Putative uncharacterized protein	0.949	0.56	0	389
32	D0CBI7	Outer membrane efflux family protein	0.952	0.629	1	499
33	D0CEV8	Periplasmic protein	0.993	0.522	0	366
34	D0C6M9	Ferrichrome-iron receptor protein	1	0.556	0	698
35	D0CB85	Porin D	0.993	0.549	0	438
36	D0C912	Autotransporter adhesin	0.995	0.702	1	2258
37	D0CD61	Putative uncharacterized protein	0.949	0.635	0	397
38	D0C7H9	FhuE receptor	1	0.652	0	721
39	D0C9Y2	Putative uncharacterized protein	0.949	0.513	0	448
40	D0C6Y1	Long-chain fatty acid transporter	0.952	0.548	0	457
41	D0C9S5	Small protein A	0.992	0.536	0	132
42	D0CFI7	Nucleoside-binding outer membrane protein	0.993	0.574	0	241
43	D0C8Y7	Lipoprotein	0.993	0.513	0	159
44	D0C985	FilF	0.949	0.678	0	641
**45**	**D0C6H3**	**β-barrel assembly machine protein BamA**	**1**	**0.539**	**0**	**841**
46	D0C8R6	FhaB protein	0.948	0.684	2	1898
47	D0C982	Pilus assembly protein	0.949	0.673	0	398
48	D0CEI5	Nuclease	0.952	0.533	0	812
49	D0C856	Putative uncharacterized protein	0.952	0.626	0	394
50	D0C5V0	Outer membrane lipoprotein blc	0.977	0.546	0	177

S. No.	UniProt ID	Protein Note	Outer membrane probability	Adhesin Probability	Trans-membrane helices	Protein Length
51	D0CEW2	Peptidyl-prolyl cis-trans isomerase	0.929	0.59	0	188
